# Monocyte-Derived Macrophages Are Impaired in Myelodysplastic Syndrome

**DOI:** 10.1155/2016/5479013

**Published:** 2016-12-15

**Authors:** Yu Han, Huaquan Wang, Zonghong Shao

**Affiliations:** Department of Hematology, General Hospital, Tianjin Medical University, Tianjin 300052, China

## Abstract

*Background*. The myelodysplastic syndrome (MDS) comprises a group of clonal hematopoietic stem cell diseases characterized by cytopenia, dysplasia in one or more of the major myeloid lineages, ineffective hematopoiesis, and increased risk of development of acute myeloid leukemia (AML). Macrophages are innate immune cells that ingest and degrade abnormal cells, debris, and foreign material and orchestrate inflammatory processes. We analyzed the role of macrophages from MDS patients in vitro.* Methods*. Macrophages were induced from peripheral blood of patients with MDS via granulocyte macrophage colony-stimulating factor (GM-CSF). Phagocytic capacity of macrophages was measured with carboxyfluorescein succinimidyl ester and fluorescent microspheres. CD206 and signal regulatory protein alpha (SIRP*α*) on macrophages were detected by flow cytometry. Inducible nitric oxide synthase (iNOS) was measured by ELISA method.* Results*. Compared with normal control group, the number of monocytes increased in MDS patients. However, the monocytes showed impaired ability to induce macrophages and the number of macrophages induced from MDS samples was lower. Further, we demonstrated that the ex vivo phagocytic function of macrophages from MDS patients was impaired and levels of reorganization receptors CD206 and SIRP*α* were lower. Levels of iNOS secreted by macrophages in MDS were increased.* Conclusions*. Monocyte-derived macrophages are impaired in myelodysplastic syndromes.

## 1. Background

Myelodysplastic syndrome (MDS) is an incurable hematological malignancy in which clonal hematopoietic stem cells proliferate and expand within bone marrow, leading to cytopenia, dysplasia in one or more of the myeloid lineages, ineffective hematopoiesis, and increased risk of development of acute myeloid leukemia (AML). Clinical studies and experimental mouse models indicate that the bone marrow microenvironment and immune system play important roles in pathogenesis of MDS [1, 2].

Macrophages are innate immune cells that are positioned throughout the body tissues, where they ingest and degrade abnormal cells, debris, and foreign material and orchestrate inflammatory processes. When monocytes migrate from the circulation and extravasate through the endothelium, they differentiate into macrophages. Monocytes and macrophages are professional phagocytic cells. The various macrophage subsets play either a protective or a pathogenic role in antimicrobial defense, allergy and asthma, autoimmunity, antitumor immune responses, tumorigenesis, metabolism and obesity, atherosclerosis, fibrosis, and wound healing [3, 4].

The role of macrophages in the pathophysiology of human malignancies has received increasing interest. In solid tumors, 5%–40% of tumor mass consists of tumor-associated macrophages (TAMs). The TAMs are now known to be important for development and progression of malignant diseases, owing to suppression of antitumor immunity. Furthermore, infiltration by TAMs is related to poor outcome in most human malignancies [5–8].

In this article, we have investigated the role of monocyte-induced macrophages in the pathogenesis of MDS.

## 2. Methods

### 2.1. Patients

Twenty-four patients diagnosed with MDS were enrolled in this study, as per the criteria of World Health Organization (WHO) (2008). The study was carried out at the Hematology Department of General Hospital, Tianjin Medical University, Tianjin, China, from September 2014 to December 2015. Basic characteristics of the patients are described in Table 1. Briefly, 1 case of refractory anemia (RA), 2 cases of RA with ringed sideroblasts (RARS), 6 cases of RA with multilineage dysplasia (RCMD), 1 case of RA with excess blasts (RAEB)1, 13 cases of RAEB2, and 1 case of 5q− syndrome were included in the study. According to the International Prognostic Scoring System (IPSS), there were 3 cases with low-, 8 cases with intermediate 1-, 11 cases with intermediate 2-, and 2 cases with high-risk MDS. There were 13 males and 11 females with median age of 58.5 (range: 16–79) years. Fifteen healthy blood donors were selected as controls, including 10 males and 5 females (median age of 46; age range: 25–56). The study was approved by the Ethics Committee of the General Hospital, Tianjin Medical University. Informed written consent was obtained from all patients or their guardians in accordance with the Declaration of Helsinki.

### 2.2. Cell Culture: Morphology and Counting

Peripheral blood mononuclear cells (PBMCs) were separated from fresh heparinized blood samples (5 mL). The PBMCs were seeded at 3–5 million cells/mL in sterile RPMI 1640 (Invitrogen, Carlsbad, CA, USA) and cultured for 7 days with the addition of granulocyte macrophage colony-stimulating factor (GM-CSF) (Huabei Pharmacy, Shijiazhuang, China). The macrophages became attached to the bottom of the culture dishes during the course of the culture. On day 7, the cells were observed under the microscope and collected for counting.

### 2.3. In Vitro Phagocytosis Assays

Normal PBMCs were labeled with 0.5 *μ*M carboxyfluorescein succinimidyl ester (CFSE; Molecular Probes, Leiden, Netherlands) and incubated with either MDS or normal human derived macrophages for 2 hours with gentle shaking at 37°C. The cells were then analyzed by fluorescence microscopy to determine the phagocytic index (PI, number of cells ingested per 100 macrophages).

### 2.4. Phagocytic Capacity of Cultured Macrophages for Detecting Fluorescent Microspheres

The fluorescent microspheres (80 *μ*L) were first incubated with 8 mL of 1% fetal bovine serum (FBS) at 37°C for 30 min and then added to a 6-well plate containing preprocessed macrophages. Each well contained 4–6 × 10^5^ macrophages and 1 × 10^7^ preconditioned fluorescent microspheres. The macrophages were incubated with microspheres at 37°C in the dark for 1.5 hours and were then harvested for flow cytometric analysis. Samples were acquired on a FACSCalibur and analyzed using CellQuest software version 3.1 (Becton Dickinson, Franklin Lakes, NJ, USA).

### 2.5. Enzyme-Linked Immunosorbent Assay (ELISA)

The level of inducible nitric oxide synthase (iNOS) in the supernatant of macrophage cultures was measured by human ELISA kit (Elabscience Biotechnology, Wuhan, China). In addition, the expression of iNOS was stimulated by lipopolysaccharide (LPS) and IFN-*γ*.

### 2.6. Measurement of Effector Proteins in Macrophages

The effector proteins of macrophages were measured in peripheral blood samples from patients with MDS and normal controls. The cells were stained with CD14, CD68, and CD206 or signal regulatory protein alpha (SIRP*α*) antibodies at 4°C for 20 min. The stained cells were then analyzed by flow cytometry. The CD206 and SIRP*α* expressed on macrophages were analyzed. The fluorophore-conjugated monoclonal antibodies (mAb) including CD14-FITC, CD68-PE, CD206-APC, and SIRP*α*-APC and relevant human isotype controls were purchased (Becton Dickinson, Franklin Lakes, NJ, USA) and used in the assays.

### 2.7. Statistical Analysis

All analyses were performed using SPSS 18.0 software (SPSS Science). Data were presented as mean ± SE. Student's *t*-test was used for two independent groups. Mann–Whitney test was used for two groups of paired data. A *p* value < 0.05 was considered statistically significant.

## 3. Results

### 3.1. Increase in the Number of Peripheral Blood Monocytes in MDS

**Figure 1:**
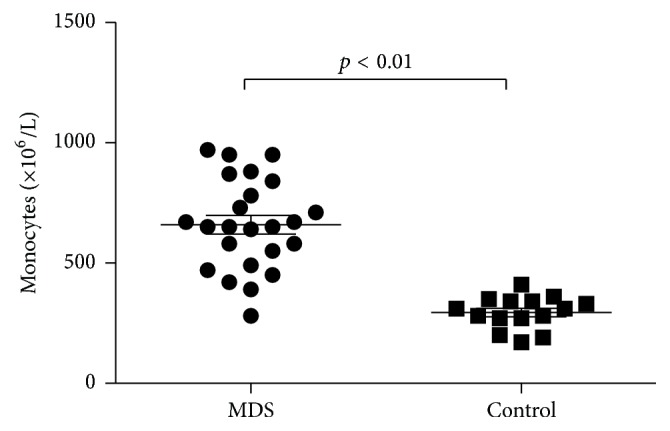
Quantity of monocytes increased in MDS patients (*p* < 0.01).

The number of monocytes was (659.2 ± 38.6) × 10^6^/L in MDS patients, while that in the controls was (294.0 ± 17.4) × 10^6^/L. The quantity of monocytes in MDS patients was higher than that in the controls (*p* < 0.01) (Figure 1).

### 3.2. Reduction in the Number of Monocyte-Induced Macrophages in MDS

**Figure 2:**
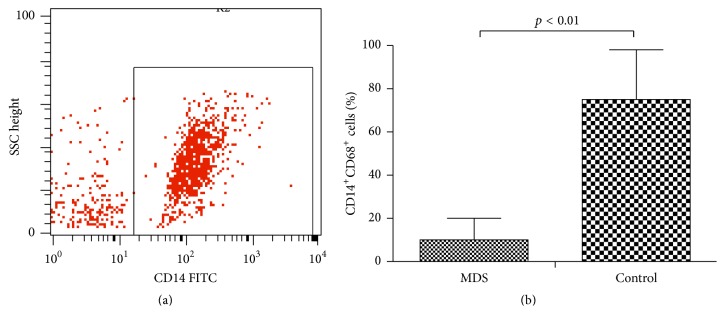
Ability of monocytes to induce macrophages was lower in MDS patients. (a) Monocyte-induced macrophages (CD14^+^) derived from peripheral blood of patients with MDS and normal controls were measured by flow cytometry. (b) Quantity of CD14^+^CD68^+^ cells decreased in MDS patients (*p* < 0.01).

The PBMCs from MDS group showed impaired capacity to induce macrophages. The macrophages were observed under the microscope and collected for subsequent experiments. The induced macrophages (CD14^+^CD68^+^) in the MDS group and normal controls were 10.06% ± 2.04% and 75.29% ± 5.94%, respectively (*p* < 0.05) (Figure 2).

### 3.3. Impairment of Macrophage Phagocytosis in MDS

The monocyte-differentiated macrophages in the MDS group showed lower phagocytic capacity than those from the normal controls by fluorescent microspheres.

**Figure 3:**
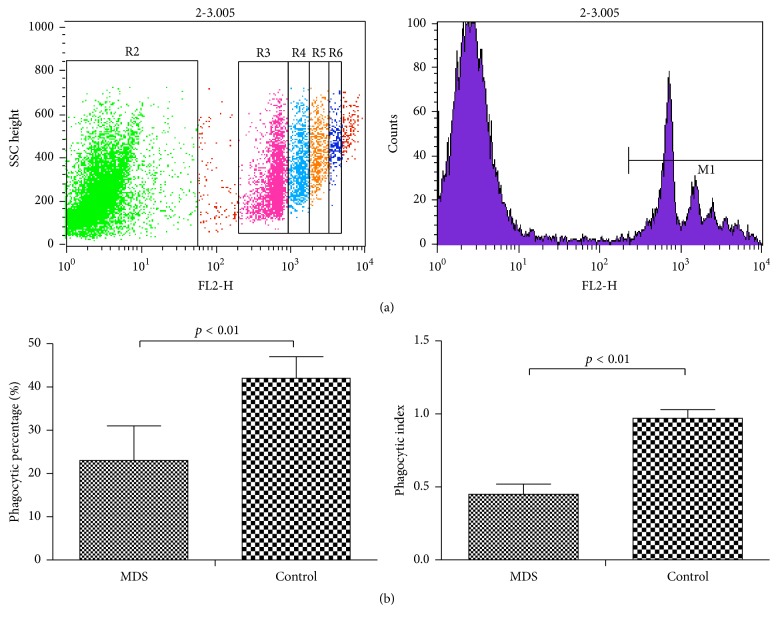
Phagocytosis of monocyte-induced macrophages as demonstrated by fluorescent microspheres. (a) Phagocytic capacity of differentiated macrophages derived from peripheral blood from patients with MDS and normal controls was tested with fluorescent microspheres by flow cytometry. In the picture, the left represents the macrophages not engulfing the fluorescent microspheres; the right represents the macrophages engulfing the fluorescent microspheres. R3 suggests that the macrophages are swallowing a fluorescent microsphere; R4 suggests that the macrophages are swallowing two fluorescent microspheres; R5 suggests that the macrophages are swallowing three fluorescent microspheres; R6 suggests that the macrophages are swallowing four fluorescent microspheres. (b) The PI and PP of monocyte-induced macrophages from MDS and normal controls are shown, respectively (*p* < 0.01).

To determine the role of macrophages, the monocyte-differentiated macrophages from patients with MDS and normal controls were evaluated. The phagocytic percentage (PP, the count of macrophages engulfing fluorescent microspheres/total macrophage cell number × 100%) of monocyte-differentiated macrophages (23.69% ± 3.22%) was significantly decreased in the MDS group compared to that in normal controls (42.75% ± 2.13%, *p* < 0.05). The PI (the total number of swallowed fluorescent microspheres/total macrophage number) was also dramatically decreased in the MDS group (0.45 ± 0.08 versus 0.92 ± 0.07, *p* < 0.05) (Figure 3).

The ability of macrophages to engulf CFSE-labeled normal PBMCs was decreased in the MDS group compared to the normal controls, as evidenced by immunofluorescence microscopy.

**Figure 4:**
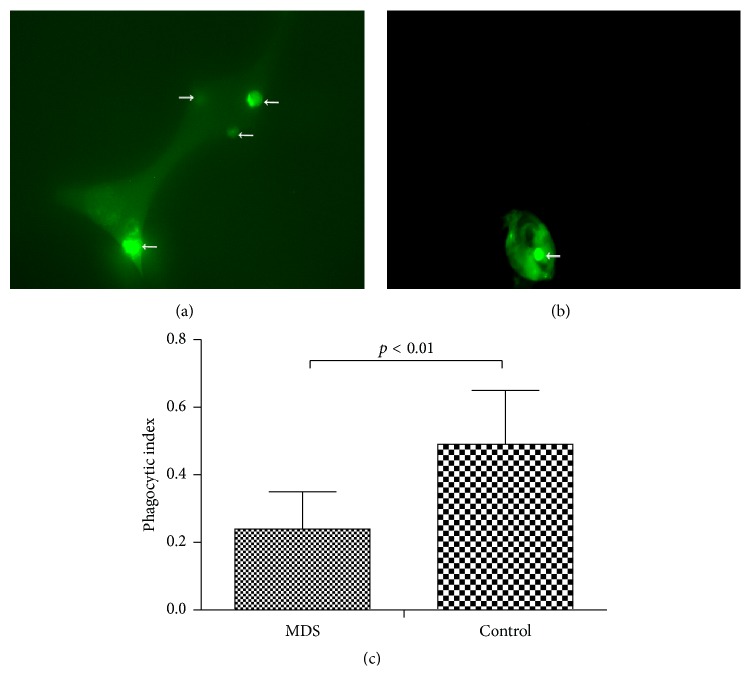
Phagocytosis of monocytes-induced macrophage as demonstrated by CFSE. CFSE-labeled normal PBMCs were incubated with monocyte-induced macrophages from either normal controls (a) or patients with MDS (b). These cells were assessed by immunofluorescence microscopy for the presence of fluorescently labeled normal PBMCs within the macrophages (indicated by arrows). (c) Phagocytic capacity of differentiated macrophages from patients with MDS and normal controls was tested by immunofluorescence microscopy. The PI of differentiated macrophages from MDS and normal controls are shown (*p* < 0.01).

We applied another method to confirm the impaired phagocytosis of macrophages in MDS patients. The CFSE-labeled normal PBMCs were incubated with macrophages from MDS patients or normal controls and then assessed for phagocytosis by immunofluorescence microscopy. The PI of macrophages in the MDS patients (0.24 ± 0.04) was significantly lower than that in the normal controls (0.48 ± 0.06, *p* < 0.05) (Figure 4).

### 3.4. Reduction of CD206 Expression on Macrophages in MDS

**Figure 5:**
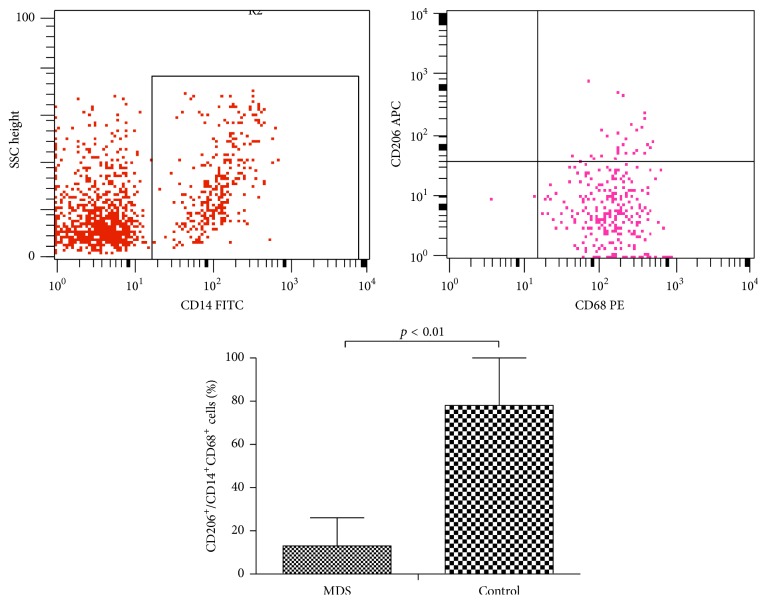
Expression of CD206 (CD206^+^/CD14^+^CD68^+^) on macrophages from peripheral blood from patients with MDS and normal controls was tested by flow cytometry (*p* < 0.01).

The expression of the macrophage mannose receptor (CD206) on macrophages in MDS patients was significantly reduced compared to that in normal controls (9.73% ± 2.59% versus 51.15% ± 10.82%, respectively; *p* < 0.05) (Figure 5).

### 3.5. Reduction of SIRP*α* Expression on Macrophages in MDS

**Figure 6:**
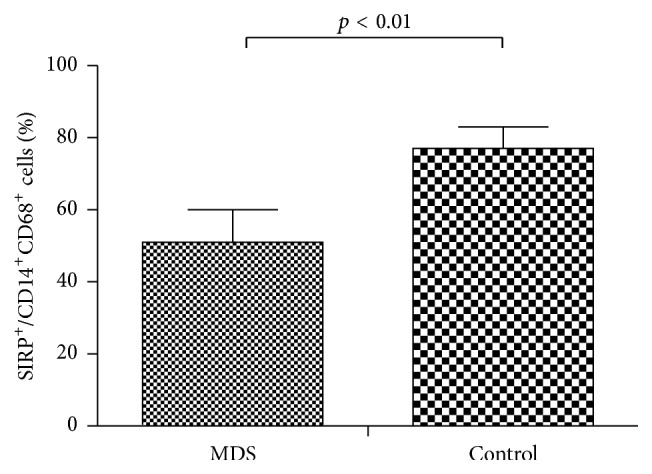
Expression of SIRP*α* (SIRP*α*^+^/CD14^+^CD68^+^) on macrophages from peripheral blood from patients with MDS and normal controls was tested by flow cytometry (*p* < 0.01).

The expression of SIRP*α* on macrophages in MDS patients was significantly reduced compared to that in normal controls (0.51% ± 0.09% versus 0.77% ± 0.06%, respectively; *p* < 0.05) (Figure 6).

### 3.6. Increased iNOS Secretion by Macrophages in MDS

**Figure 7:**
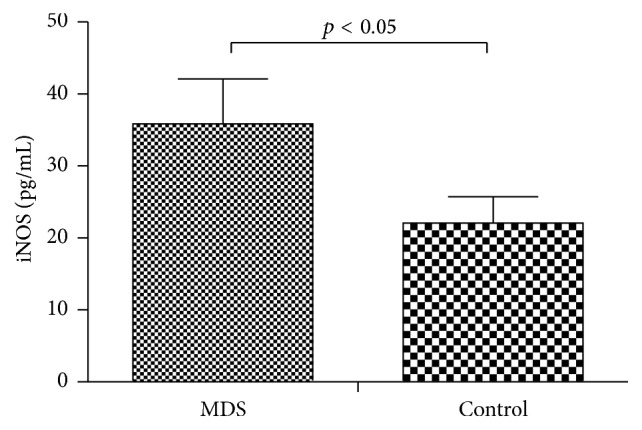
Comparison of levels of iNOS in the supernatant of macrophage cultures from either MDS patients or normal controls, as measured by ELISA (*p* < 0.05).

The level of iNOS in the supernatant of macrophage cultures from MDS patients was increased compared to that in normal controls (35.87 ± 6.25 pg/mL versus 22.05 ± 3.67 pg/mL, respectively; *p* < 0.05) (Figure 7).

## 4. Discussion

Cancer development is a multistep process involving sequential mutations in oncogenes and tumor suppressor genes of normal cells, resulting in the transformation into a tumor cell [9]. Subsequent uncontrolled cell division typically progresses from precancerous lesions to malignant tumors. However, in addition to alterations in tumor cells, the microenvironment is essential for driving the progression of malignancies. The microenvironment surrounding the tumor mass contains excessively proliferating tumor cells along with several host components, including stromal cells, an expanding vasculature, and a characteristic inflammatory infiltrate associated with the constant tissue remodeling. Experimental data demonstrate the role for these individual components in promoting tumor growth and progression. Specific examples include endothelial cells [10], macrophages [11], and cancer-associated fibroblasts [12]. It appears that most components of the immune system are endowed with potential dual functions. For example, immune cells exhibit the ability to reject tumors on one hand by producing antitumor cytokines, thereby directly destroying tumor cells. On the other hand, these immune cells can be recruited by tumor cells to help in progression of cancer. Moreover, immune cells that have infiltrated a tumor mass can create a microenvironment producing cytokines, chemokines, growth factors, and angiogenic factors that promote tumor progression [13].

Traditionally, macrophages have been described as tumoricidal cells. Macrophages have a pleiotropic biological role that includes antigen presentation, target cell cytotoxicity, removal of debris and tissue remodeling, regulation of inflammation, induction of immunity, thrombosis, and various forms of endocytosis. Increasing evidence indicates that macrophages can also adopt a protumor phenotype in both primary tumors and metastases, as they can promote growth, angiogenesis, metastasis, and immunosuppression [3, 4, 14]. In the setting of tumors, TAMs have a range of functions with the capacity to affect diverse aspects of neoplastic tissues including angiogenesis and vascularization, stroma formation and dissolution, and modulation of tumor cell growth (enhancement and inhibition). When activated, they can induce neoplastic cell death (cytotoxicity and apoptosis) and/or elicit tumor destructive reactions through alterations of the tumor microvasculature [5, 6].

In our study, we observed that although the number of monocytes in majority of the MDS patients was increased, the macrophages derived from MDS monocytes were fewer and exhibited impaired phagocytosis. This suggested that the ability of abnormal monocytes to develop into normal macrophages was inhibited in MDS patients. Moreover, when abnormal MDS clonal cells arise, the macrophages fail to phagocytose them. In addition, macrophages and monocytes could be partly progenies of MDS clone in most cases, which leads to bone marrow protumor microenvironment.

We observed that the levels of CD206 and SIRP*α* were decreased in MDS patients compared with those in normal controls. CD206, which is a macrophage mannose receptor, enables the macrophage to bind to microorganisms and internalize them during the process of phagocytosis [15, 16]. SIRP*α* is an immunoglobulin superfamily protein that binds to the protein tyrosine phosphatases SHP-1 and SHP-2 through its cytoplasmic region. CD47, another immunoglobulin superfamily protein, is a ligand for SIRP*α*, with the two proteins constituting a cell-cell communication system (the CD47-SIRP*α* signaling system) [17, 18]. This might explain the observation that macrophages originating from MDS patients could not bind to or phagocytose “tumor cells” as competently as those from normal controls. Therefore, we may speculate that the impaired macrophages from MDS patients might be a result of the biological behavior of tumor growth and progression.

In this study, we found that iNOS expression was upregulated in MDS patients, compared to normal controls. Human carcinomas are associated with upregulation of iNOS, which is otherwise generally not expressed in normal (noncancerous) tissues, with the exception of the kidney, brain, and placenta [19]. Human carcinomas exhibiting high levels of iNOS expression include those in stomach, liver, and lung. This synthase is involved in many physiological and pathological processes. Moreover, its expression is closely related to the biological behavior of tumor growth, progression, metastasis, and prognosis. A key function of iNOS is the enzymatic conversion of arginine to generate a locally high concentration of nitric oxide (NO). From a tumorigenic perspective, the iNOS-mediated increase in NO supports cancer development [20]. The above evidence indicates that iNOS contributes to the development of MDS.

## 5. Conclusions

In this study, we explored the role of macrophages in the pathogenesis of MDS by inducing the monocytes to become macrophages. Compared with normal controls, the macrophage phagocytosis activity in MDS patients was abnormal. The expressions of recognized receptors CD206 and SIRP*α* were lower in macrophages in MDS patients, but the level of iNOS was increased. These results suggested that macrophages in MDS patients could not recognize, phagocytose, and kill the MDS clonal cells. Our study provides a new insight for the research of macrophages in MDS patients, while it also offers a new therapeutic strategy targeting macrophages.

## Figures and Tables

**Table 1:** Clinical characteristics of MDS patients.

Case	Sex/age	Diagnosis	Cytogenetics	IPSS
1	Female/61	5q−	Good	Int-1
2	Female/79	RAEB-2	Good	Int-2
3	Female/68	RAS	Good	Int-1
4	Female/49	RAEB-2	Good	Int-1
5	Male/25	RCMD	Good	Int-1
6	Female/62	RAEB-2	Good	Int-2
7	Male/57	RAEB-2	Good	Int-2
8	Male/42	RAS	Good	Low
9	Female/70	RAEB-2	Good	Int-2
10	Male/30	RAEB-2	Int	High
11	Female/29	RAEB-2	Poor	High
12	Male/58	RAEB-1	Poor	Int-2
13	Male/58	RAEB-2	Good	Int-2
14	Female/69	RAEB-2	Good	Int-2
15	Male/76	RAEB-2	Good	Int-2
16	Male/59	RAEB-2	Good	Int-2
17	Male/61	RAEB-2	Good	Int-2
18	Female/60	RAEB-2	Good	Int-2
19	Male/49	RA	Good	Low
20	Male/16	RCMD	Int	Int-1
21	Male/50	RCMD	Good	Int-1
22	Female/41	RCMD	Int	Int-1
23	Female/64	RCMD	Good	Low
24	Male/69	RCMD	Int	Int-1

## Data Access

The author should be contacted for data requests.
